# Continuous hemodiafiltration as a rescue therapy for patients with cardiopulmonary failure caused by enterovirus-71: a retrospective observational study in a PICU

**DOI:** 10.1186/s12879-019-4519-9

**Published:** 2019-10-21

**Authors:** Chunxia Wang, Yun Cui, Yan Zhu, Fei Wang, Qunfang Rong, Yucai Zhang

**Affiliations:** 1 Department of Critical Care Medicine, Shanghai Children’s Hospital, Shanghai Jiao Tong University, No.355 Luding Road, Shanghai, 200062 Putuo District China; 2 Institute of Pediatric Critical Care, Shanghai Jiao Tong University, Shanghai, 200062 China

**Keywords:** Enterovirus71, Hand, foot and mouth disease, Cardiopulmonary failure, Continuous veno-venous hemodiafiltration, Mortality, Child

## Abstract

**Background:**

Hand, foot and mouth disease (HFMD) remains a burdensome health issue in mainland China. Enterovirus71 (EV-A71) is the main pathogen of severe HFMD. Continuous hemofiltration improves fluid overload, restores kidney function and alleviates inflammatory reactions. The aim of the present study was to evaluate the effects of continuous veno-venous hemodiafiltration (CVVHDF) on severe HFMD caused by EV-A71(EV-A71-HFMD) in a pediatric intensive care unit (PICU).

**Methods:**

A retrospective observational study was performed in a tertiary university PICU from January 2012 to December 2016. Children with severe EV-A71-HFMD complicated by cardiopulmonary failure were included. The patients were divided into a CVVHDF group and a conventional therapy (control) group (non-CVVHDF). The demographics, characteristics, and outcomes between the groups were collected and analyzed.

**Results:**

Twenty-nine patients with severe EV-A71-HFMD were enrolled. The 28-day mortality was 17.6% (3/17) in the CVVHDF group and 33.3% (4/12) in the non-CVVHDF group, with no statistical significance between the two groups (*P* = 0.403). The median interval between CVVHDF initiation and PICU admission was 6 (4,8.5) hrs, and the median duration of CVVHDF was 48 (36, 64) hrs. The left ventricular ejection fraction (LVEF) and cardiac index (CI) in the CVVHDF group were improved after treatment. The plasma levels of catecholamines and renin-angiotensin-aldosterone system (RAAS) substances in the CVVHDF group were significantly decreased after treatment. The decreased catecholamines and RAAS substances included adrenalin (169.8 [145.5, 244.6] vs. 148.0 [109.0, 208.1] ng/L, *P* = 0.033), dopamine (152.7 [97.0, 191.1] vs. 96.0 [68.0, 160.9] ng/L, *P* = 0.026), angiotensin II (185.9 [125.2, 800.0] vs. 106.0 [90.8, 232.5] ng/L, *P* = 0.047), aldosterone (165.7 [94.0, 353.3] vs. 103.3 [84.3, 144.3] ng/L, *P* = 0.033), and renin (1.12 [0.74, 3.45] vs. 0.79 [0.52, 1.25] μg/L/h, *P* = 0.029),

**Conclusions:**

CVVHDF reduced the levels of catecholamines and RAAS substances and improved cardiovascular function. Continuous hemodiafiltration may represent a potential therapy in patients with severe EV-A71-HFMD complicated with cardiopulmonary failure.

## Background

Hand, foot and mouth disease (HFMD) has become a major global health issue, and HFMD in China accounted for 87% (9.8 million/11.3 million) of all HFMD cases reported to the World Health Organization (WHO) from 2010 to 2014 [1]. Cardiopulmonary failure is the main cause of mortality in children with HFMD under the age of 3 years [2]. In 2012, a large outbreak of HFMD occurred over a wide area in China, and enterovirus71 (EV-A71) was responsible for 60.4% of HFMD inpatients and 88.5% of severe cases with cardiopulmonary failure [3]. According to the China Guidelines for the diagnosis and treatment of severe HFMD due to EV-A71 [4], severe HFMD with cardiopulmonary failure caused by EV-A71, referred to as stage 3–4 EV-A71-HFMD, remains a life-threatening and challenging disease in a pediatric intensive care units (PICUs) in China.

Continuous renal replacement therapy (CRRT) is available for pediatric critically ill patients. Moreover, CRRT is suitable for renal replacement, fluid overload (FO) management, homeostasis stabilization, toxin removal, and proinflammatory cytokine release in infants and young children [5, 6]. Children with severe HFMD develop rapidly progressing sympathetic hyperactivity, pulmonary edema, and cardiopulmonary failure [7, 8]. Inappropriate fluid resuscitation and acute kidney injury (AKI) may lead to FO in cases of severe HFMD. Limited studies have observed that hemofiltration is be an efficient rescue treatment in children with severe HFMD [9–11].CRRT has been empirically used in patients with severe EV-A71-HFMD with cardiopulmonary failure since 2012 in our PICU. Our previous studies indicated that continuous hemofiltration effectively reduced the inflammatory response and improved organ dysfunction in patients with severe sepsis [12, 13] or secondary hemophagocytic lymphohistiocytosis [14]. Thus, we suspected that hemofiltration could be a potential therapy for severe EV-A71-HFMD complicated by cardiopulmonary failure.

Tachycardia and systemic hypertension are symptoms of ulminant EV-A71-related HFMD and are considered to be a sympathetic storm [15]. An excited sympathetic tone with a catecholamine surge might contribute to extremely high systemic vascular resistance, cardiac dysfunction, and passive pulmonaryedema [16]. In the present study, we retrospectively analyzed the medical records of patients with severe EV-A71-HFMD complicated by cardiopulmonary failure. The benefits of continuous veno-venous hemodiafiltration (CVVHDF) on mortality and clinical parameters were analyzed. We speculated that catecholamines and renin-angiotensin-aldosterone system (RAAS) substances may play key roles in the process of cardiopulmonary failure caused by EV-A71 in patients with stage 3 or 4 HFMD. Therefore, we measured the levels of epinephrine, dopamine, rennin, angiotensin II and aldosterone in patients with stages 3 or 4 HFMD.

## Methods

### Study design

A retrospective observational study was performed in patients with severe EV-A71-HFMD complicated with cardiopulmonary failure admitted to the PICU at Shanghai Children’s Hospital between January 2012 and December 2016. According to whether the patients received CVVHDF during PICU hospitalization, the patients were divided into a non-CVVHDF group, who underwent conventional therapy, or a CVVHDF group, who underwent conventional therapy plus CVVHDF. The study was conducted in accordance with the ethical principles of the Declaration of Helsinki (and subsequent revisions) and the current standards for observational studies. This study was approved by the Ethics Review Committee, Children’s Hospital of Shanghai/Shanghai Children’s Hospital, Shanghai Jiao Tong University and conducted in accordance with the provisions of the Declaration of Helsinki (Approval No. 2016R010-F01). The need for informed consent to participate was waived because we used deidentified retrospective data.

### Patients

Diagnosis and staging were performed according to the clinical therapy expert consensus on severe cases caused by EV-A71. Patients with severe EV-A71-HFMD complicated with cardiopulmonary failure were diagnosed as having stage 3 or 4 [4]. The symptoms of patients in stage 3 or 4 include increased heart and respiratory rates, cold sweats, cold extremities, mottled skin, increased blood pressure, tachycardia (bradycardia wasalso occasionally seen), tachypnea, cyanosis, cough with pink foamy or bloody sputum, hypotension and ultimately cardiovascular collapse [4].

The inclusion criteria were as follows: (1) patients aged 1 month to 14 years old; (2) patients with severe stage 3 or 4 EV-A71-HFDM, defined by the clinical therapy expert consensus on severe cases caused by EV-A71 (4); and (3) patients who were EV-A71 IgM or RT-PCR positive. Patients who required cardiopulmonary resuscitation and died within 24 h after admission were excluded.

The conventional management for HFDM was performed according to the 2008 Guidelines for the diagnosis and the expert consensus on the rescue and treatment of severe cases caused by EV-A71 [4, 17]. The physiological fluid requirement was 60–80 ml/kg/day in the absence of deliberate diuresis. Patients with shock were resuscitated with normal saline 10–20 ml/kg/time over 30 min while administering vasoactive agents [4, 17]. The hemodynamic change in stage 3 was characterized by high dynamicity and high resistance. Milrinone was used with a loading dosage of 50–75 μg/kg. The maintenance dose was 0.25–0.75 μg/kg/min. The total duration of the infusion perioddid not exceed 72 h. Phentolamine (1–20 μg/kg/min) or sodium nitroprusside (0.5–5 μg/kg/min) was initiated at a low dose and gradually increased to an appropriate dose level to control blood pressure to a level below that of constituting severe hypertension at the corresponding age if necessary. When hypotension manifested in patients with stage 4 EV-A71-HFDM, positive inotropic agents and vasopressors, such as dopamine at 5–20 μg/kg/min, norepinephrine at 0.05–2 μg/kg/min, adrenalineat 0.05–2 μg/kg/minute and dobutamine at 2.5–20 μg/kg/min were used. If patients had suffered from nosocomial infection or were supported by mechanical ventilation for more than 3 days, antibiotics were used.

### Continuous hemofiltration

The indications for CVVHDF initiation in our study included an FO > 10%[FO = (CVVHDF initial weight-PICU admission weight)/PICU admission weight× 100%], acute kidney injury (AKI), or unstable hemodynamics, such as cardiogenic shock shock and multiple organ dysfunction [18, 19]. For patients with severe EV-A71-HFMD presenting with refractory cardiovascular disorder, CVVHDF as an empirical adjuvant therapy combined with conventional treatment was administered to maintain stable hemodynamics. Patients who suffered from severe coagulopathy disorder (international normalized ratio [INR] > 3.0 or platelet count< 10 × 10^9^/L; *n* = 6) or a history of biofilm or hemofilter allergy (*n* = 1) or patients without parental consent for CRRT(*n* = 5) were treated with conventional therapy and included in the non-CVVHDF group.

CVVHDF was performed ata flow rate of 35–50 mL/kg/hr. for both ultrafiltrate and dialysate using a PRISMA or PRISMA flex blood purification machine and Gambro PRISMA M60 membrane hemofilter equipped with an AN69 (Gambro Renal Products, Meyzieu, France). Vascular access was obtained with an 8F central venous catheter (GamCath; Gambro, Colombes, France) in the right internal jugular or femoral vein according to the patient’s body weight. Blood flow was set at a constant 4–6 mL/kg/min to achieve a filtration fraction of 25–35%. The replacement fluid was prepared according to the modified Ports formula and contained Na^+^ 130 mmol/L, K^+^ 4 mmol/L, HCO_3_^−^ 28 mmol/L, Ca^2+^ 1.5 mmol/L, Mg^2+^ 3.2 mmol/L, Cl^−^ 109 mmol/L, and glucose 0.2 g/L. The pre- to postdilution ratio was 1:2. The rate of replacement fluid was 35 ml/kg/h (Qf) or 10–15 ml/min (Qd).The filter circuit was pretreated with saline that contained 5000–10,000 IU/L unfractionated heparin. During CVVHDF, unfractionated heparin was used with at aninfusion rate of 5–20 U/kg/h. The activated partial thromboplastin time (APTT) was detected every 4–6 h, and the transmembrane pressure was modified between 50 and 120 mmHg. The dose of heparin was regulated to maintain APTT at 1.5–2-fold the normal value. The hemofilter was changed every 24 h or when clotted. The patients with severe coagulation disorders (APTT > 80 s or INR > 3.0), a biofilm allergic reaction, or difficult venin catheter access were managed with conventional therapies, as in the control group. The implementation of CVVHDF for cardiovascular indications was fully explained, and the parents of the patients provided written informed consent prior to CVVHDF initiation.

In the present study, the indications for the weaning of CVVHDF included the following: (1) a normal heart rate and blood pressure; (2) serum lactate < 2 mmol/L; and (3) a urine output more than 1 ml/kg/h and FO < 10%. The primary endpoints were as follows: (1) ameliorated hyperthermia; (2) improved pulmonary edema and pulmonary effusion; (3) a urine output of > 1 ml/kg/h; and (4) a normal blood pressure. Continuous hemofiltration was terminated when the following conditions occurred: (1) the children developed severe bleeding or uncontrollable hemorrhaging; or (2) symptoms were not obviously improved after 72 h. The assessments for initiation and weaning, as well as the effects of CVVHDF, were performed by an attending intensivist in our PICU.

### Data collection and definitions

All data were retrieved from the hospital database system. Variables were defined prior to data collection and were entered in a standardized format during data collection. The collected data included demographic data (such as age, sex, and body weight), details of the initial presentation on admission, clinical features for EV-A71-induced HFMD, length of PICU stay, vital signs (temperature, heart rate, and blood pressure), cardiac parameters (left ventricular ejection fraction, LVEF; and cardiac index, CI), the ratio of the partial pressure of oxygen in arterial blood (PaO_2_) to the inspired oxygen fraction (FiO_2_) (PaO_2_/FiO_2_) before and after CVVHDF, as well as 28-day mortality, duration of mechanical ventilation, and duration of vasoactive agent administration. Cardiac ultrasound was performed by a specialized cardiologist. Laboratory data, including pH, creatinine (Cr), alanine transaminase (ALT),serum lactate (Lac), cardiac troponin I (cTnI) and creatine kinase-MB (CK-MB), as well as catecholamine-like substances, including adrenalin, dopamine, renin, angiotensin II, and aldosterone, were obtained from the computerized hospital medical database. Biochemical indexes were collected at admission and after 3 days in the non-CVVHDF group. Biochemical indexes were collected before CVVHDF initiation and after CVVHDF for 3 days in the CVVHDF group.

### Statistical analysis

The data were analyzed using SPSS (v. 22.0) (SPSS Inc., Chicago, IL). All the variables were tested for normal distribution using the Kolmogorov-Smirnov test. Continuous variables with abnormal distributions were summarized as medians (IQRs). The Mann-Whitney *U* test was used to compare the continuous variables with abnormal distributions. The *chi*-square test was used to compare the categorical data. A value of *P* < 0.05 was considered statistically significant.

## Results

### Baseline characteristics of patients with SevereEV-A71-HFMD with cardiopulmonary failure

Twenty-nine patients with severe EV-A71-HFMD complicated with cardiopulmonary failure were included. All patients received mechanical ventilation (29/29). Twelve patients were managed with conventional therapy, and 17 patients were treated with conventional therapy plus CVVHDF. There was no significant difference in age, sex, body weight, temperature, heart rate, blood pressure, LVEF, CI or the use of vasoactive agents between the non-CVVHDF group and the CVVHDF group. There was no significant difference in the incidence of AKI and FO between the non-CVVHDF group and the CVVHDF group (2 cases vs. 4 cases, *P* = 0.653; 3 cases vs. 6 cases, *P* = 0.694; respectively) **(**Table 1**)**.

**Fig. 1:**
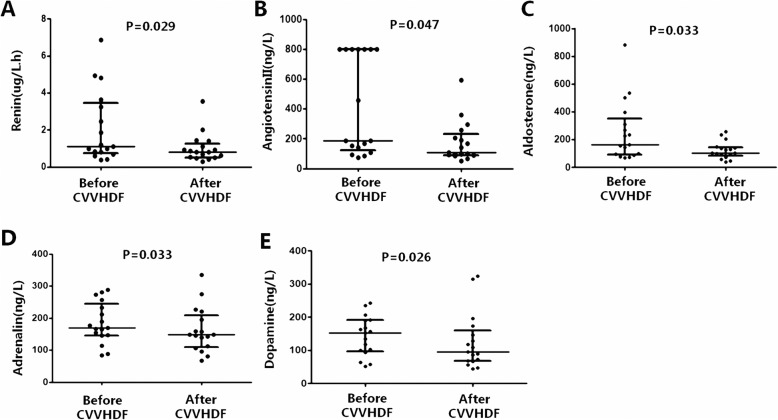
Effect of continuous veno-venous hemodiafiltration (CVVHDF) on catecholamines and renin-angiotensin-aldosterone system (RAAS) substances. **a** renin, **b** angiotensin II, **c** aldosterone, **d** adrenaline, **e** dopamine

**Table 1:** Baseline characteristics of patients with severe HFMD caused by EV71 in the non-CVVHDF group and the CVVHDF group

Characteristics	Non-CVVHDF group (*n* = 12)	CVVHDF group (*n* = 17)	*P*
Age, month	21.0 (8.3, 23.8)	23.0 (12.5, 24.0)	0.372
Male, n	8	10	0.717
Severity			0.979
Stage 3, n	7	10	
Stage 4, n	5	7	
Body weight, kg	10.6 (8.3, 14.4)	11.0 (10.0, 14.4)	0.565
Body temperature, °C	39.7 (39.4, 39.8)	39.7 (39.3, 40.0)	0.518
Heart rate, bpm	188 (171, 195)	187 (178, 198)	0.982
LVEF, %	38 (26, 44)	37 (24, 47)	0.773
CI, L/min/m^2^	2.89 (2.23,3.63)	2.73 (1.92, 3.95)	0.642
Systolic blood pressure, mmHg	132.0 (76.0, 150.5)	139 (102, 155)	0.116
Acute kidney injury, n	2	4	0.653
Fluid overload, n	3	6	0.694
Mechanical ventilator, n	12	17	/
Vasoactive agents			
Milrinone, n	7	7	0.462
Phentolamine, n	1	0	0.414
Sodium nitroprusside, n	4	2	0.198
Dopamine, n	4	6	0.913
Dobutamine, n	9	13	1.000
Norepinephrine, n	1	3	0.622
Adrenaline, n	2	4	1.000

*LVEF* Left ventricular ejection fraction, *CI* Cardiac index. Continuous variables were summarized as the median (IQR)

### Outcomes and laboratory indexes of patients in the non-CVVHDF group and the CVVHDF group

The levels of pH (*P* = 0.842), PaO_2_/FiO_2_ (*P* = 0.757), lactate (*P* = 0.249), Cr (*P* = 0.757), ALT (*P* = 0.894), cTnI (*P* = 0.877) and CK-MB (*P* = 0.308) were not significantly different between the two groups. The median interval between CVVHDF initiation and PICU admission was 6 (4,8.5) hrs. The duration of vasoactive agent administration and the length of stay in the PICU were significantly decreased in the CVVHDF group compared with the non-CVVHDF group (*P* = 0.024, *P* = 0.030, respectively; Table 2). Moreover, the total 28-day mortality of severe EV-A71-HFMD was 4/12 in the non-CVVHDF group and 3/17 in the CVVHDF group, without a significant difference (*P* = 0.403, Table 2). The duration of mechanical ventilation had a decreased tendency in the CVVHD group compared with the non-CVVHDF group, but the difference was not significant (*P* = 0.063, Table 2).

**Table 2:** Outcomes and laboratory indexes in patients with severe HFMD caused by EV71 in the non-CVVHDF group and the CVVHDF group

Characteristics	Non-CVVHDF group (n = 12)	CVVHDF group (n = 17)	*P*
Biochemical data			
PH	7.21 (6.94, 7.37)	7.23 (7.07, 7.31)	0.842
PaO_2_/FiO_2_, mmHg	134 (112.5, 157.8)	135 (101.5, 160)	0.757
Lac, mmol/L	4.35 (3.20, 5.80)	3.5 (2.8, 3.9)	0.249
Cr, μmol/L	58.0 (28.5, 79.8)	48 (28, 77)	0.757
ALT, IU/L	36.0 (19.8, 63.3)	35 (13, 70)	0.894
cTnI, μg/L	0.16 (0.06, 0.38)	0.12 (0.06, 0.39)	0.877
CK-MB, IU/L	35 (21, 73)	37 (26, 112)	0.308
Outcomes			
Duration of mechanical ventilation, day	14.5 (11.5, 20.3)	8.0 (5.5, 17.5)	0.063
Duration of vasoactive agents, day	7.5 (6.0, 10.0)	5.0 (4.5, 7.0)	0.024
Stay in PICU, day	23 (17, 27)	12 (9, 22)	0.030
28-day death, n	4	3	0.403

*PaO*_*2*_*/FiO*_*2*_ The ratio of the partial pressure of oxygen in arterial blood (PaO_2_) to the inspired oxygen fraction (FiO_2_), *Lac* Lactate, *Cr* Creatinine, *ALT* Alanine transaminase, *cTnI* Cardiac troponin I, *CK-MB* Creatine kinase-MB. Continuous variables were summarized as the median (IQR)

### Effects of CVVHDF

The median duration of CVVHDF was 48 (36, 64) hrs. Fever (*P* = 0.001), heart rate (*P* = 0.001), systolic blood pressure(*P* = 0.003), LVEF (*P* = 0.017) and CI (*P* = 0.002) were significantly improved after treatment in the CVVHDF group (Table 3). The levels of blood lactate were significantly decreased in the CVVHDF group (3.50 [2.75, 3.90] vs. 1.3 [0.95, 1.90] mmol/L, *P* = 0.001, Table 3). Moreover, the levels of adrenalin (169.8 [145.5, 244.6] vs. 148.0 [109.0, 208.1] ng/L, *P* = 0.033), dopamine (152.7 [97.0, 191.1] vs. 96.0 [68.0, 160.9] ng/L, *P* = 0.026), renin (1.12 [0.74, 3.45] vs. 0.79 [0.52, 1.25] μg/L·h, *P* = 0.029), angiotensin II (185.9 [125.2, 800.0] vs. 106.0 [90.8, 232.5] ng/L, *P* = 0.047), and aldosterone (165.7 [94.0, 353.3] vs. 103.3 [84.3, 144.3] ng/L, *P* = 0.033) were significantly decreased after CVVHDF (Table 3, Fig. 1). In the non-CVVHDF group, fever (*P* = 0.001) and heart rate (*P* = 0.001) were significantly improved, and the levels of blood lactate were significantly decreased after 72 h of conventional treatment (4.35 [3.20, 5.80] vs. 2.2 [1.93, 3.75] mmol/L, *P* = 0.014, Table 3). Moreover, the pH level improved (7.21 [6.94, 7.37] vs. 7.31 [7.21, 7.38], *P* = 0.015). However, the levels of adrenalin, dopamine, renin, angiotensinII and aldosterone did not change after 3 days of treatment in the non-CVVHDF group (*P* = 0.190, *P* = 0.678, *P* = 0.872, *P* = 0.215, and *P* = 0.730, respectively, Table 3).

**Table 3:** Changes of characteristics of severe HFMD caused by EV71 in the non-CVVHDF group and the CVVHDF group

Variables	Non-CVVHDF group (n = 12)			CVVHDF group (n = 17)			*P”*
	1st day (*A1*)	3rd day (*A2*)	*P*	Before CVVHDF (*B1*)	72 h after CVVHDF (*B2*)	*P’*	
Vital signs							
Heart rate, bpm	188.0 (170.8, 194.8)	142.0 (130.3, 157.0)	0.001	187.0 (178.0, 197.5)	134.0 (122.5, 147.0)	0.001	0.143
Body temperature, ^o^C	39.7 (39.4, 39.8)	38.0 (37.3, 38.5)	0.001	39.7 (39.3, 40.0)	36.8 (36.4, 37.3)	0.001	0.001
Systolic blood pressure, mmHg	132.0 (76.0, 150.5)	121.5 (103.5, 140.3)	0.854	139 (101.5, 155.0)	102.5 (86.5, 115.7)	0.003	0.001
Cardiac parameters							
LVEF, %	38 (26, 44)	41 (29, 46)	0.204	37 (21, 47)	55 (21, 62)	0.017	0.184
CI, L/min/m^2^	2.89 (2.23, 3.63)	3.11 (2.22, 3.73)	0.621	2.73 (1.92, 3.95)	4.77 (1.92, 5.75)	0.002	0.199
cTnI, μg/L	0.16 (0.06, 0.38)	0.08 (0.04, 0.15)	0.032	0.12 (0.06, 0.39)	0.02 (0.01, 0.48)	0.365	0.325
CK-MB, IU/L	35.0 (20.5, 72.8)	33.0 (19.0, 64.3)	0.240	37.0 (25.5, 112.0)	78.0 (39.0, 110.5)	0.298	0.008
Organ function indexes							
Blood glucose, mg/dL	9.85 (6.98, 12.18)	7.9 (6.95, 10.35)	0.311	9.4 (7.9, 11.3)	6.1 (5.7, 11.8)	0.118	0.057
Lac, mmol/L	4.35 (3.20, 5.80)	2.2 (1.93, 3.75)	0.014	3.50 (2.75, 3.90)	1.3 (0.95, 1.90)	0.001	0.005
pH	7.21 (6.94, 7.37)	7.31 (7.21, 7.38)	0.015	7.23 (7.07, 7.31)	7.39 (7.37, 7.44)	0.001	0.041
Cr, μmol/L	58.0 (28.5, 79.8)	61.0 (54.5, 119.5)	0.121	48.0 (28.0, 77.0)	38.0 (20.5, 72.0)	0.517	0.023
ALT, IU/L	36.0 (19.8, 63.3)	63.0 (27.0, 140.8)	0.048	35.0 (12.5, 70.0)	38.0 (26.0, 52.0)	0.227	0.170
Catecholamine							
Adrenalin, ng/L	153.6 (127.0, 184.4)	145.4 (85.8, 175.1)	0.190	169.8 (145.5, 244.6)	148.0 (109.0, 208.1)	0.033	0.223
Dopamine, ng/L	127 (82.4, 156.5)	133.4 (83.0, 171.7)	0.678	152.7 (97.0, 191.1)	96.0 (68.0, 160.9)	0.026	0.319
RAAS substances							
Renin, μg/L.h	2.35 (1.49, 4.76)	2.90 (1.30, 4.80)	0.872	1.12 (0.74, 3.45)	0.79 (0.52, 1.25)	0.029	0.003
AngiotensinII, ng/L	348.4 (131.5, 773.0)	565.4 (210.9, 773.3)	0.215	185.9 (125.2, 800.0)	106.0 (90.8, 232.5)	0.047	0.001
Aldosterone, ng/L	211.2 (119.8, 362.6)	281.9 (167.9, 349.0)	0.730	165.7 (94.0, 353.3)	103.3 (84.3, 144.3)	0.033	0.001

*LVEF* Left ventricular ejection fraction, *CI* Cardiac index, *cTnI* Cardiac troponin I, *CK-MB* Creatine kinase-MB, *Lac* Lactate, *Cr* Creatinine, *ALT* Alanine transaminase. Continuous variables were summarized as the median (IQR). *P* indicates A1 vs. A2; *P′* indicates B1 vs. B2; *P″* indicates A2 vs. B2

### Six-month follow-up assessments

During a 6-month follow-up, 1 patient in the CVVHDF group died due to central respiratory failure. One patient in the CVVHDF group was discharged with difficulty swallowing but recovered. The other patients were alive without comorbidities at the time of follow-up.

## Discussion

Patients with severe EV-A71-HFMD complicated with cardiopulmonary failure have a high risk of mortality. In the present study, we demonstrated the benefits of CVVHDF in improving cardiopulmonary function and decreasing the levels of catecholamines and RAAS substances, which were associated with a decreased tendency of 28-day mortality in severe EV-A71-HFMD patients. To our knowledge, this study is the first report regarding the impact of CVVHDF on EV-A71-HFMD.

A previous report indicated that the mortality associated with EV-A71-induced severe HFMD in the PICU was 30.4% (7/23) during the 2010 ongoing outbreak in the Shanghai region [3]. A recent study showed that the mortality rate of severe HFMD was 21.3% (19/89) in the PICU at the Children’s Hospital of Chongqing Medical University of China from June 2015 to September 2016 [20], suggesting an improvement in the management of severe HFMD in China in recent years. In the present study, the 28-day mortality rate of severe EV-A71-HFMD was lower in the CVVHDF group than in the non-CVVHDF group (3/17 vs. 4/12, *P* = 0. 403), implying that the application of CVVHDF might contribute to the decreased tendency in mortality. In our previous study, CVVHDF showed clinical benefits in the management of severe sepsis, sepsis-associated hemophagocytic lymphohistiocytosis, and sepsis-associated liver injury in our PICU [12–14, 21]. The current study focused on CVVHDF empiric therapy for severe EV-A71-HFMD, as CVVHDF has been a recommend therapy for severe pediatric EV-A71-HFMD complicated by cardiopulmonary failure in the 2018 Guideline for HFMD in China [4]. This warrants further research using well-designed prospective trials with a large population.

Our previous study on severe EV-A71-HFMD indicated that the involved organ systems affected by EV-A71 included the central nervous system, the respiratory system, and the cardiovascular system [22]. In the present study, 29 patients with EV-A71-HFMD displayed high heart rates and high lactate levels on PICU admission, and all patients received mechanical ventilation for respiratory failure or cardiogenic pulmonary edema. Limited studies found that EV-A71-infected patients with autonomic nervous system dysregulation and/or pulmonary edema had high levels of norepinephrine (NE) and epinephrine (EP) [23], and plasma NE gradually increased with the aggravation of the disease [24]. In the present study, we observed that the blood levels of catecholamines, including adrenalin and dopamine, decreased after treatment in the CVVHDF group but not in the non-CVVHDF group. To date, the effects of continuous hemofiltration on the removal of catecholamines are controversial. Bellomo and colleagues [25] found no significant amounts of catecholamines in the ultrafiltrate of critically ill patients with AKI who received CRRT. Inconsistently, the ultrafiltrate from patients with heart failureproduced a positive inotropic response potentially attributable to catecholamines [26]. Furthermore, Siebeck M et al. [27] indicated that the catecholamine concentration was significantly decreased after continuous hemofiltration. Given that adrenalin (molecular weight: 219) and dopamine (molecular weight: 153) are “small” molecules, we speculated that adrenalin and dopamine may be removed directly by CVVHDF. Because EV-A71-induced catecholamine storms play a crucial role in cardiopulmonary failure [28], the reduction of catecholamines by CVVHDF might contribute to the improvement of cardiopulmonary failure in patients with severe EV-A71-HFMD.

To date, there is little information available regarding RAAS involvement in EV-A71-HFMD. Whether CVVHDF removed RAAS substances resulting in an improvement in cardiopulmonary failure requires further investigation. To our knowledge, we are the first to report that the levels of renin, angiotensin II, and aldosterone were significantly decreased in patients in the CVVHDF group. Several studies indicated that the RAAS blockade reduced cardiovascular morbidity and mortality, and angiotensin converting enzyme inhibitor therapy was used in patients with left ventricular dysfunction [29, 30]. Therefore, we speculated that RAAS components might contribute to the protective effects of CVVHDF on cardiopulmonary failure in severe EV-A71-HFMD patients.

Our study has several limitations. First, only 17 patients received CVVHDF in our retrospective study, which affected the power of the conclusion. Second, an analysis of the filtered solution is necessary to identify the role of CVVHDF in removing catecholamines and RAAS substances. Third, the difference in the CK-MB levels after treatment between the non-CVVHDF and CVVHDF groups needs further investigation in a large population. Nevertheless, our results are noteworthy because CVVHDF showed a tendency to improve 28-day mortality in severe EV-A71-HFMD patients. This result warrants further research using well-designed prospective trials based on a large population.

## Conclusions

CVVHDF represents a potential therapy in patients with severe EV-A71-HFMD complicated with cardiopulmonary failure; CVVHDF improved cardiovascular function associated with reduced levels of adrenaline, dopamine, as well as RAAS substances including rennin, angiotensin II, and aldosterone.

## Data Availability

Our present study was a retrospective observational study. All the data were obtained from the medical records of patients. All the data generated or analyzed during this study are available from the corresponding author upon reasonable request.

## Abbreviations

AKI: Acute kidney injury
ALT: Alanine transaminase
APTT: Activated partial thromboplastin time
CI: Cardiac index
CK-MB: Creatine kinase-MB
Cr: Creatinine
CRRT: Continuous renal replacement therapy
cTnI: Cardiac troponin I
CVVHDF: Continuous veno-venous hemodiafiltration
EV-A71: Enterovirus71
EV-A71-HFMD: Severe HFMD caused by EV-A71
FO: Fluid overload
HFMD: Hand, foot and mouth disease
INR: International normalized ratio
Lac: Lactate
LVEF: Left ventricular ejection fraction
PaO_2_/FiO_2_: The ratio of the partial pressure of oxygen in arterial blood (PaO_2_) to the inspired oxygen fraction (FiO_2_)
PICU: Pediatric intensive care unit
